# Willing to Donate? A Quantitative and Thematic Analysis of Attitudes to Deceased Organ Donation in Different Circumstances

**DOI:** 10.1111/hex.70765

**Published:** 2026-08-05

**Authors:** Weiyi Tan, Janet Cruse, Dominic Summers, Zoë Fritz

**Affiliations:** ^1^ School of Clinical Medicine, Addenbrooke's Hospital University of Cambridge Cambridge UK; ^2^ Department of Surgery, Level E9 Addenbrooke's Hospital University of Cambridge Cambridge UK; ^3^ THIS Institute (The Healthcare Improvement Studies Institute) University of Cambridge Cambridge UK

## Abstract

**Introduction:**

The UK public's willingness to donate, knowledge of, and concerns about different organ donation scenarios are not known.

**Methods:**

A validated questionnaire was developed based on a literature review of the ethical issues associated with uncontrolled Donation after Circulatory Death (uDCD). Participants read three scenarios (Donation after Brain Death [DBD], controlled, and uncontrolled Donation after Circulatory Death [cDCD and uDCD]) relating to being asked about organ donation (with clear explanations of what was involved) in a randomised order. Each vignette was followed by Likert and open‐ended questions. Quantitative and thematic analyses were undertaken.

**Results:**

Five hundred and five UK participants, representative of ethnicity, gender, and age (range 18–79) were recruited via *Prolific* and completed the survey. Participants were likely/willing to donate in 366/504 (73%) for DBD; 336/505 (67%) for cDCD; and 368/503 (73%) for uDCD. Ninety‐six per cent believed uDCD is already part of donation practice. Qualitative analysis revealed altruism as the most dominant theme. Participants did not want organs wasted, wanted to respect loved ones' wishes, and thought organ donation would provide comfort for the bereaved. For uDCD 71% agreed/strongly agreed that prioritising the chance to donate, including invasive techniques, was most important, noting the value of time for discussion; 93% thought they would need less than 4 h to decide. A small proportion of people did not support organ donation in any situation, largely for bodily integrity and religious reasons.

**Conclusions:**

In this questionnaire survey, the UK public broadly supported donation in different circumstances, including uDCD, which the majority think already happens.

**Patient or Public Contribution:**

A Patient and Public Involvement (PPI) group of more than 30 people have been involved in a larger research project on uDCD. Two of these, both bereaved relatives of potential donors, were consulted about the design and content of the questionnaire, and provided written and oral feedback, leading to changes in wording and style; the overall research proposal, nature of questions and recruitment was supported. One of these, JC, became a core part of the research team, attending meetings with other team members and contributing to the critical analysis and write up of the research; in particular she independently read the entire dataset searching for contradictory or outlying examples and agreed that the themes reported were representative, was involved in the final drafting of the manuscript, and is a co‐author.

**Abbreviations**
cDCD: controlled donation after circulatory death
DBD: donation after brain death
NHSBT: National Health Service Blood and Transplant
NRP: normothermic regional perfusion
ODASICA: organ donation after sudden irreversible cardiac arrest
PPI: patient and public involvement
uDCD: uncontrolled donation after circulatory death

## 1 Introduction

Organ donation is important, emotive, and underperformed. There is a shortage of organs for transplantation in the UK with over 7000 patients currently waiting for a kidney [1]. Despite around 300,000 deaths per year in adults under the age of 80, and 30,000 out‐of‐hospital cardiac arrests attended by the ambulance service [2], there are only around 1500 deceased donors annually in the UK [3].

One reason for this mismatch is that organ retrieval currently occurs only in ‘controlled’ settings: donation following brain death (DBD) or expected death after treatment withdrawal in Intensive Care Units (ICU) (controlled Donation after Circulatory Death, cDCD). There is no current pathway in the UK for organ donation after sudden irreversible cardiac arrest (currently called ‘uncontrolled’ DCD or uDCD) [4, 5].

Anecdotally, the failure to provide a donation after out‐of‐hospital cardiac arrest pathway is distressing for the bereaved. A public engagement group convened by our team revealed some believed uDCD donation already occurred in the UK, and all felt that it should. Successful uDCD programmes are already well established in France and Spain [6, 7, 8, 9], and are underway in other countries [10, 11].

There are logistical and ethical challenges associated with uDCD [12, 13], which have rendered it controversial [14]. After confirming irreversible arrest and death (via arterial monitoring and a 5‐min ‘hands‐off’ period) [15, 16], organs must be rapidly preserved to avoid damage from blood and oxygen deprivation [7]. In France and Spain, normothermic regional perfusion (NRP) is used to preserve abdominal organs after death, yielding excellent transplant outcomes. However, this provokes ethical debates about restoring in situ circulation after death [17]. Some argue it respects patient preferences; others question whether the public sees it as excessively risky or invasive [18].

Additionally, unlike in controlled DCD, where families have more time to process loss, uDCD involves shorter timeframes. Public opinion on its acceptability is key. Previous studies (under opt‐in systems and before NRP) suggest people may be as or more willing to discuss donation soon after Emergency Department death than after ICU brain death [19, 20]. However, no studies have assessed public understanding or views on uDCD specifically. Our primary aim was to evaluate how willing the general public were to consider donation in different circumstances (brain death, expected death following ICU withdrawal, and out‐of‐hospital cardiac arrest). Our secondary aims were to understand their reasons for these views and to understand public views on both organ preservation after death and communication with families about donation.

## 2 Methods

Members of the public were recruited to answer a questionnaire consisting of three vignettes and a series of closed and open questions. Ethics approvals were received from the Cambridge University Psychology Research Ethics Committee (PRE.2024.015), and the research was co‐designed with our PPI colleagues, one of whom (JC) joined as a core member of the research group.

Construct validity was initiated by drawing on the existing literature, and building on studies by DuBois et al. (2009) [19], Volk et al. (2010) [20], and Bedenko et al. (2016) [21]. Working with these as a baseline, we wrote three vignettes (see Box 1), asking the participant to imagine that they were being approached to consider organ donation for their relative in three different scenarios: following brain death, following withdrawal of treatment, and following sudden irreversible cardiac arrest. Participants were informed about the legal framework for opting in and out [22, 23] (see Box 2), and then presented with the vignettes in a random order, to reduce order effect ‐ we anticipated that participants might be anchored by the first vignette they encountered and so mitigated against this through randomisation of which study was read first, with all participants reading and responding to all three vignettes. Each vignette was followed by both Likert Questions on a 3–5‐point scale and open‐ended questions, in order to provide data on the frequency of views, and the reasons for them. Participants were asked their views on organ preservation and communication with staff. At the end of the questionnaire (to avoid priming) participants were asked about their own views on organ donation in general and their own intention to donate.

BOX 1 **Vignettes used in study (appeared in random order).**

1 **Donation after brain death**

Imagine that your family member was in an accident, was taken to the hospital, and placed on life support. Even though their heart is still beating, two senior doctors (consultants) both carry out a series of tests which show that the brain is dead and will never recover. This is called brain death; the family member has died. They advise that the machines that are currently on to keep the heart pumping and the lungs moving should be stopped. A different member of clinical staff asks you whether you think the family member would have wanted to donate their organs.

**Donation after withdrawal of life support**

Imagine that your family member was in an accident, was taken to the hospital Intensive Care Unit and placed on life support, which means that a machine is breathing and pumping blood for them. Although there is still some brain activity on the tests, they are in a coma, and are not responding to any stimulation and their heart is not working well on its own. Despite the doctors' best efforts, your family member does not improve, and you are told that they will never recover enough to come off life support and so it should be turned off, so that they can die peacefully. A different member of clinical staff asks you whether you think the family member would want to donate their organs after the life support machine is turned off and they have died.

**Donation after cardiac arrest out of hospital**

Imagine that your family member's heart stopped suddenly out of hospital. Cardiopulmonary Resuscitation (CPR) was attempted immediately, and the paramedics arrived quickly and transferred you and your family member to hospital, continuously attempting resuscitation. Despite optimal and prolonged CPR attempts, the doctors conclude that the cardiac arrest was irreversible and that your family member has died (cardiac death). A different member of clinical staff explains that it may be possible for your family member to donate their organs. In order for this to happen, a small tube will be put into the blood vessels in the top of their leg, and a machine will be set up to keep blood flowing to the kidneys and the liver (but not the brain or heart), in order to preserve some organs and give you time to think about whether your family member would want to donate their organs.

BOX 2 **Current practice of deceased organ donation in England (exactly as written to participants).**

The law was changed in 2019 to what is known as ‘deemed consent’ – if you haven't explicitly ‘opted‐out’ from organ donation, then it is assumed that you would want to be a donor, unless your family or next of kin object.

You can still actively ‘opt in’ (via the organ donor register, your GP or when you apply for your driving licence). If you do this, then clinicians do not need the consent of the family before proceeding to try to retrieve your organs, although they will still always try to talk with family members about the process.

The questionnaire was assessed for content validity with a panel of experts (the Blood and Transplant team, transplant co‐ordinators, and transplant surgeons) who were asked to consider whether the questionnaire items measured all relevant domains of the constructs established from the existing literature and previous questionnaires. Face validity was assessed iteratively, initially with 10 non‐medical volunteers going through an observed cognitive interviewing or ‘thinking aloud’ completion of the questionnaire [24]. Participants were asked to explain what they understood the questions to mean, and what they were thinking while they wrote the answers. In response to this, changes were made to improve readability and understanding, and the process was repeated three times with 24 people in total. The questionnaire can be found in Appendix A.

A sample of the general public representative for age, gender, and ethnicity [25] was recruited using prolific.com https://www.prolific.co/academic‐researchers. Prolific is an online research platform designed primarily for recruiting participants for academic research [26]. Several studies have shown that data gathered from Prolific is high quality in terms of attention and depth [25, 27, 28]. It was estimated that the survey would take 15 min to complete, and participants were paid £4 (£12/h) for their time. Participants provided consent before completing the survey and could withdraw at any time.

### 2.1 Data Was Stored on Qualtrics

Quantitative data were analysed using SAS 9.4, Cary, NC. and anonymised qualitative data were imported to NVivo and thematically analysed by authors using the framework method [29], with initial codes having been determined from the literature review, and with additional emerging codes identified and refined. Two researchers conducted the coding; meetings took place with a multi‐disciplinary research team (WT, ZF, DS, and JC). Where there was discordance, codes were further defined and refined to ensure consistency. All text and codes were reviewed by at least two authors.

## 3 Results

A total of 505/529 participants completed the survey in August 2024 (95% response rate, demographic data in Table 1). Seventy‐eight per cent said they would be definitely or likely to be themselves willing to donate, which is consistent with national data which reports this at 80%, suggesting that our sample reflected national attitudes to donation more generally [30]. Question completion rate was excellent with 502/505 participants completing all but the ranking question (465 responses), including comprehensively completing free text boxes with relevant information suggesting high‐quality data [28]. A total of 79,354 words were written by the 505 participants.

**TABLE 1:** Demographic data of participants.

Demographic data	*n*	%
Gender		
Female	246	49
Male	259	51
Ethnicity		
White	423	84
Asian	42	8.4
Black	18	3.6
Mixed	10	2
Other	10	2
Student status		
Yes	44	8.8
No	386	76.7
No data	73	14.5
Employment status		
Full time	216	42.9
Part‐time	71	14.1
Unemployed	16	3.2
Due to start job	4	0.8
Not in paid work	88	17.5
Other	16	3.2
No data	92	18.3

*Note: N* = 505. Participants were on average 46.8 years old (SD = 15.7, range 18–79).

Quantitative and qualitative results are integrated below, with representative quotes used to illustrate the reasoning participants gave for their answers; Table 2 presents main qualitative themes with illustrative quotes and Table 3 presents uDCD‐specific qualitative themes.

### 3.1 Willingness to Donate in General

**FIGURE 1:**
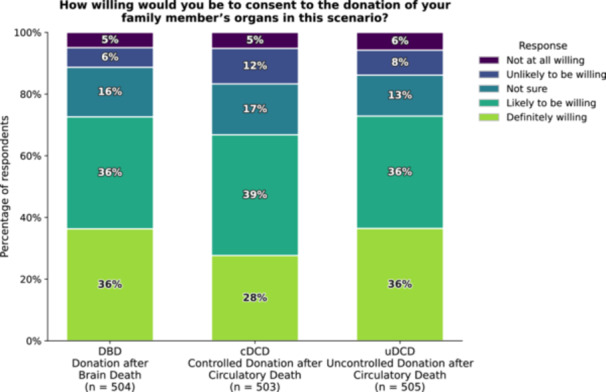
Willingness to donate in different donation scenarios (DBD *n* = 504; cDCD *n* = 503; and uDCD *n* = 505).

Support for all organ donation scenarios was consistently high with participants likely or definitely willing to donate in DBD (366/504; 73%), cDCD (336/505; 67%), and uDCD (368/503; 73%) (Figure 1).

#### 3.1.1 Willing to Donate: Altruistic Motivations, Respecting Wishes, and Aiding Bereavement

The primary reason for this was altruism. Many participants mentioned this in at least one of their answers: “*organ donation after death is a profound act of altruism, offering the possibility to save or significantly improve the lives of others. The idea that even in death, a person can contribute to the well‐being of others is a powerful motivator for me” (P413)*. Altruism was considered both a moral and pragmatic duty.

Knowing the deceased person's wishes was considered of greater significance than the (living) family's wishes: “*the main factor here is the person's own wishes before their death, not that of their family” (P99)*.

An additional strong theme was providing comfort for the bereaved: *“I think, in an odd way, that this is a beautiful scenario and could give the family some peace of mind, knowing that lives can be saved” (P328)*.

#### 3.1.2 Unwilling to Donate – Consent, Bodily Integrity, and Religion

A small proportion of people (27/505 or 5%) did not support organ donation (not at all willing or unlikely to be willing) in any context. Their reasons fell into three broad categories: not knowing the deceased individual's wishes; wanting to maintain bodily integrity; religious reasons, and sometimes an interplay between the last two: *“it is part of my religion to not allow the body to be tampered with in any way” (P447)*.

While the overall quantitative results were quite similar across all scenarios, there were specific qualitative themes – both positive and negative – for each scenario.

### 3.2 Willingness to Donate in Donation After Brain Death

In donation after brain death, most participants found certainty in the irreversibility of brain death reassuring: *“brain death equals death, quite simply” (P434).* However, the concept of brain death was not universally understood. Several participants conflated brain death and persistent vegetative state, being concerned about overall quality of life: *“I would rather see my loved ones help save others through the donation of their organs than to keep them alive but in a vegetative state” (P428)*. Other participants expressed insights into the emotional and cognitive dissonance about death that might occur: *“while this would be hard, as their heart is still beating, I understand that they are actually dead, but it would be difficult” (P6)*.

### 3.3 Willingness to Donate in Controlled Donation After Circulatory Death

In cDCD, many participants recognised the futility of further treatment and wanted someone to benefit from organs where possible: *“if there is no hope of recover [sic], don't want them to suffer, and can help others that do have a chance” (P61)*. Of the minority that were not willing to donate in this scenario, the most common theme was concern about the uncertainty of prognosis, and the potential of their loved ones recovering*: “I think people cling on to the hope that their loved one will recover if their brain is active (P241)”*. People were also concerned about feelings of guilt in what they perceived to be their involvement in making a decision to withdraw treatment: “*turning off the life support would be a much more difficult decision than donating the organs” (P165)*.

### 3.4 Willingness to Donate in Uncontrolled Donation After Circulatory Death

**FIGURE 2:**
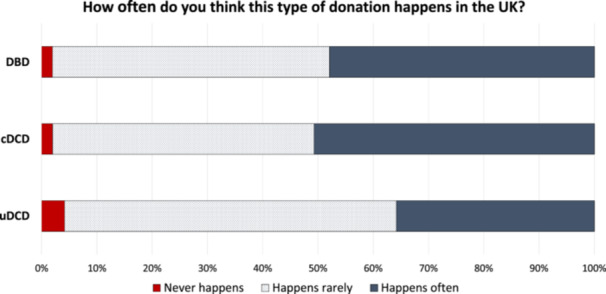
Perceived frequency in the UK of each type of donation scenario (DBD *n* = 505; cDCD *n* = 503; and uDCD *n* = 505).

Our explicit quantitative finding – that participants were as willing to donate in uDCD as DBD ‐ is better understood in the context of the qualitative explanations. Respondents perceived a greater certainty that the patient is dead (in contrast with other scenarios, where withdrawal of life support needed to occur): *“As it was a cardiac arrest I feel their death was as natural as can be. There is no hope for recovery and I would agree to donate their organs” (P151)*. When explicitly asked, participants reported that they were comfortable with the process of cannulation and perfusion of the organs, which we had explained in detail: “*I have no problems with this. The relative is already pronounced dead, and maintaining some blood flow to preserve the organs just makes logistical sense” (P99)*. However, of the minority not willing to donate in this scenario, the most common theme related to the procedures: *“I don't like the idea of inserting the tube to keep organs pumping blood around and worry about the impact this would have on my loved one after their passing” (P10)*. A few participants also mentioned concerns about the timing of the decision in response to this answer: *“it must be very difficult to come to terms with such a quick event and perhaps a person needs to come to terms with the death before considering organ donation” (P493)*. A large majority of participants thought uDCD was already part of donation practice in the UK with only 21/505 (4%) believing that uDCD never happened (see Figure 2).

**TABLE 2:** Main qualitative themes with illustrative quotes.

Major theme	Sub theme	Illustrative quotes
Altruism	General altruism	*“I just feel that organ donation is amazing and something that people don't want to talk about. It gives children and adults a chance of life. It's a beautiful thing to do.” (P178)* *“I 100% would want to donate my family members' organs. It would give an opportunity for somebody to create and gain their life back. And part of my family member would live on within them. I wouldn't want my family member to be living a life they were completely unhappy with or in pain or found distressing but to live on in a happy way through someone else!” (P176)* *“I feel organ donation, whether my own or a loved ones is an ultimate selfless act – we should do our best to help those still living and in need.” (P225)* *“I am completely for organ donation to help save lives!” (P19)*
	Ethical or moral duty	*“I think in this scenario it is also morally right to donate their organs as it can help other people who might need an organ.” (P201)* *“I concur that morally and ethically, donating would be a hugely positive outcome in contrast to a tragedy, as there are long lists of patients awaiting organs for many conditions, and a lack of organs/donations.” (P443)* *“I believe if you can help someone it is your duty to.” (P364)*
	Pragmatic altruism	*“The deceased has no need for their organs so why not help another in need unless deceased had asked not to.” (P64)* *“They will no longer be of any use to them.” (P301)* *“I can't see any reason why the organs should not be passed on to people who would be able to survive by receiving them. They are no longer required by my family member, so what is there to gain by not passing them on to someone so that they can continue to enjoy life with their family?” (P271)*
	Extreme altruism	*“I think it would be selfish not to consider organ donation unless the deceased left very specific instructions to NOT have their organs donated.” (P64)* *“Since his brain is dead and will never recover, I believe it will be inhumane not to help others who are alive and need the specific organs to survive.” (P374)*
Bodily integrity		*“I would imagine most people would be so distressed that they would not want to think of their loved one being cut up and part of them being given away.” (P147)* *“I would probably want my loved one's body left alone after death.” (P391)* *“I would feel a strong connection to my family member and want them to remain pure and whole.” (P151)*
Comfort for the bereaved		*“Organ donation is so important. The ability to save several lives can be such a gift and make give comfort to families of the donor.” (P311)* *“I would want some good to come from such a tragic happening.” (P187)* *“I would find it comforting that organs from a loved one can be used to save other lives.” (P88)* *“Giving someone else a chance to live in a great way through donation would be the most perfect thing to do after grieving. Remembering that a part of them still live on would make me super happy.” (P498)*
Deceased person's wishes		*“It would depend on the family members wishes.” (P41)* *“Would be ok donating it as long as I know that specific family member would have wanted it.” (P51)* *“I wouldn't be willing to give permission because of a lack of written/verbal consent from that family member to me, therefore I would think that I would be acting immorally if I acted otherwise.” (P157)* *“I already know the wishes of my next of kin/close family members & they are all of the view that they would want to donate if possible.” (P114)*
Definition of death	Brain death	*“I think if the person is brain dead there is no coming back from that.”(P400)* *“Once someone is brain dead they are gone and it is just a body made up of organs.” (P430)*
	Circulatory death	*“As there is still some brain activity i feel that this is wrong.” (P162)* *“As long as there is some activity in the brain, my view would be that the person is not dead and that there could be a small chance of them regaining consciousness.” (P121)* *“Unwilling due to their still being brain activity and heart still working even if not well.” (P175)*
	Certainty of death in uDCD	*“My family member has passed and there is not a single chance of bringing them back then yes I would instantly donate their organs to help others.” (P176)* *“Death has occurred and is irreversible however if organs can be retrieved and benefit someone else some comfort can be taken from the situation.” (P408)* *“As it was a cardiac arrest I feel their death was as natural as can be. There is no hope for recovery and I would agree to donate their organs.” (P151)*
	Emotional and cognitive dissonance about death	*“But the heart is still active so I think most people will be hesitant to consider donating at this point. At this point it might be hard to realise that the family member is dead.” (P221)* *“It is sometime difficult to accept a persons death when they are still breathing even though it is a machine that is keeping them breathing.” (P291)* *“This one is really hard as you wouldn't want to miss the opportunity to donate, though this feels harder as it's something being done to their body before they're truly dead.” (P164)*
Emotional distress	General	*“You are struggling to understand and come to terms with what has happened and may not be open for such difficult conversations.” (P206)* *“It's hard to say with certainty under such shocking emotional circumstances.” (P175)* *“It seems like a really difficult situation and a difficult decision to make in a very distressing environment.” (P55)*
	Conflict about grieving and donation	*“If I had just lost a family member, the last thing on my mind would be organ donation.” (P25)* *“Where you are having to deal with the loss of a loved one it is much more difficult to immediately switch to the practicalities of donation.” (P148)* *“Not been selfish but at this point i'd be grieving and likely unable to think straight.” (P316)*
	Hypothetical vs. reality	*“I think I'd need to experience this first hand in order to know how I would feel at the time with heightened emotions.” (P39)* *“it's incredibly difficult to answer because the death of a loved one it's such an impossible thing for as to imagine, I really have no idea how I would react in the scenario and I would capable of saying anything.” (P86)* *“It's very hard to imagine the pain of this decision. I hope I would be strong enough to agree to it and not be hanging onto the hope that my relative would recover.” (P29)*
	Insensitive/inhumane/intrusive	*“The thought of my loved one being cut up upsets me.” (P117)* *“It's distressing to consent to them being ‘prodded' after they have died.” (P321)* *“This sounds a bit intrusive when someone should be left dead.” (P490)*
	Shock factor (uDCD)	*“I would be in a state of shock and, to be honest, I think this would be the last thing on my mind.” (P110)* *“This sort of death is sudden and a huge shock and the families may have trouble even accepting the death and therefore not be ready to say yes to donation of organs.” (P286)* *“As it's a sudden unexpected event I can imagine people might not be ready to think about donation.” (P58)*
Public awareness		*“I think a lot of people are against the idea of organ donation as they don't fully understand what it entails and means for them in practice.” (P17)* *“No matter what the situation, if my family member wanted to be an organ donor, they would be on the register. As staff are asking if they would give consent to take organs, that means they are not on the register and therefore I would not go against my family members' wishes.” (P355)* *“I don't think all are aware that they need to opt out but unsure how other people will feel.” (P252)* *“Older people may not know how to go online or if you have a hectic lifestyle, it's probably not high on your to‐do list. If it was made law, that everyone had to be registered one way or another, there would be no need for family to make difficult decisions after a loved one has passed.” (P355)*
Religious reason		*“Purely for religious reasons. As a muslim, we believe that organs shouldn't be donated after death and the body should be buried as is.” (P5)* *“Religious grounds not permitted to remove parts.” (P42)* *“I'm not willing to donate my organs after my death for spiritual reasons.” (P144)*
Trust in medical professionals	Trust	*“I trust Doctors and Professionals. I would always assume they are performing what is best for the patient, and other patients. If they chose to keep a relatives body on life support, to preserve organs, even with minimal chance of recovery. I would understand.” (P78)* *“It would be good to know the organs are in safe, professional hands.” (P146)* *“I would tend to trust the opinion of the doctors.” (P219)* *“With guidance and information from medical staff I would feel at ease with the decision as their organs would be healthy and would save another person's life.” (P258)*
	Mistrust	*“Will doctors fight to save me if they know they will get my organs?” (P190)* *“I believe that family members are being pressured by medical people at this point in time when this should not be the case.” (P377)* *“Based fully on the trust in doctors and tests that are both fallible.” (P72)* *“It is a finely balanced decision. I would worry about the danger of mistakes and abuse of the system of writing people off prematurely too secure donation.” (P191)*
cDCD specific	Quality of life	*“I again, would not hang to the empty vessel of a loved one, and would much rather donate in the hope it would help someone else.” (P193)* *“This person is no longer alive, while they're still breathing it's hardly a life and so they can help others.” (P92)* *“If there is no chance of recovery I'd want the organs to be used to potentially save the life of someone else.” (P119)*
	Uncertainty about prognosis	*“Whilst in the majority of cases brain activity doesn't always mean someone will wake from a coma that rare possibility is always a piece of hope I would cling to and would be hesitant to give up life support.” (P245)* *“I would need to be absolutely sure that my relative has no hope of recovery.” (P191)* *“There are too many examples of people ‘miraculously’ coming back to life after long comas. I would want to wait a while longer…” (P434)*
	Withdrawal of treatment	*“It's more emotive because there is still brain activity. People might have guilt about ‘switching off’.” (P58)* *“The action of turning off life support feels proactive and actively choosing to determine their death and end their life, potentially resulting in ‘what if?’ scenarios in the future. I would feel reticent about turning off the life support and in turn organ donation.” (P305)* *“If they are never going to come off life support then I would sadly agree to turning off the life support and donating their organs.” (P430)*

**TABLE 3:** Qualitative themes from uDCD‐specific questions with illustrative quotes.

		Illustrative quotes
Discussions with family	Preference for treating doctor vs. specialist team	*“Both professional people have insight to the process an both would provide valuable information.” (P180)* *“I wouldn't mind who spoke to me about organ donation. The conversation is important.” (P216)* *“I would be more connected to the team who treated them.” (P392)* *“The specialist donation team who may be better informed/experienced in dealing with the situation.” (P408)*
	Conflict of interest concerns	*“I want the doctor treating my family to be focussed on their recovery not to view them as a donor.” (P79)* *“A specialist donation team might want to push too hard for me to agree to donation. A doctor will give a more balanced view.” (P341)* *“I think talking to a team who specialise in organ donation would be better as they had nothing to do with trying to save them. There would be a one step removed from the traumatic situation.” (P356)*
	Importance of good communication	*“I would like to be as informed as possible.” (P195)* *“It's important for me to be talked to by the doctors so I can understand what is happening and protect my loved one's wishes.” (P59)* *“Even if I am willing and agree to donate my loved one's organs. I'd still like to be talked through it to help process the fact they are gone, and what will happen. It'd also feel more humane for them to talk to the family first. It's polite.” (P243)*
	Importance of obtaining consent	*“Although I agree with enabling the organs to be preserved until donated, I do still believe in being asked for consent to continue.” (P246)* *“I think you should always discuss this with the family as I don't think most people are aware it is now an opt out system and the family are most likely to know their beliefs and it would be unethical to take organs someone hasn't consented to taking.” (P38)* *“I feel I would have a right to know what is happening and to ensure that the consent is clear.” (P149)*
	Nature of communication	*“Clear and professional communication is vital in these kinds of circumstances.” (P315)* *“As long as it is done with compassion I would agree to being asked. I would probably know my relatives' wishes.” (P503)* *“I think that it is important to have open dialogue at such a traumatic time in the family's life.” (P69)*
Acceptability or organ preservation techniques	Cannulation acceptable	*“The person is probably already full of tubes and needles so one more shouldn't make a difference.” (P379)* *“There is nothing wrong with putting a tube in the leg to give the family time to consider things. It seems like a minor procedure without ‘desecrating’ the body.” (P221)* *“This way of collecting organs doesn't seem so invasive. And if it means this would still help other people I'm sure many would be open to the idea.” (P378)* *“I don't see how anyone can object to the tube in the leg, since it's doing no harm to the dead or dying person.” (P331)*
	Concerns about cannulation	*“I think this sounds painful for the person, and intrusive.” (P341)* *“The idea of putting in a tube just to keep organs fresh for donation is fairly horrible.” (P391)* *“I wouldn't want to put my loved one through what could be a painful procedure.” (P190)* *“If I decided not to proceed I would not have wanted my loved one's body to have been treated in that way unnecessarily.” (P481)*
	Concerns about perfusion of organs	*“That makes me squeamish and I don't like the pumping the blood around just those organs. If the doctor said the family member was dead, I would just donate the organs, not keep some organs alive.” (P165)* *“The idea of pumping blood to maintain certain organs vital seems a bit disturbing to me.” (P358)* *“It's a very emotive subject. The idea of blood still flowing round the required organs makes it feel like the person is still alive in part but I accept they are not.” (P175)*
Prioritisation of organ preservation vs. family consent	Organ preservation techniques providing time	*“I am happy for tubes to be added to the dead body as this is a minor action and it would give me more time to make a decision.” (P487)* *“Because my family member has already died and inputting the tube is only a minor thing and gives me and my family time to decide on organ donation.” (P487)* *“For me, if inserting the tube meant I could stay with the person for a few hours to say goodbye while the donations were organised, that would be a bonus.” (P379)* *“To put a tube in the body does not seem overly invasive and there is a massive benefit of time and organ donation that goes along with that, the benefits outweigh the doing nothing and waiting approach.” (P349)*
	Importance of maintaining viability	*“Better to do something with minimal harm at the risk of it being unnecessary in the end, compared to not doing something that rules a really positive thing out completely until it's too late.” (P72)* *“I think it's a very simple thing that doesn't affect the body that much but the impact of doing it is great. It's the difference between being able and not able to donate organs. It would give me time to make a decision rather than having to rush bc of decomposition setting in.” (P27)* *“Not much point granting my permission if the organs are already useless.” (P240)* *“This happened to me and I wasn't asked if they would have wanted their organs donating. It is a big regret of mine.” (P224)*
	Prioritisation of family consent	*“Consent is paramount prior to this actually happening.” (P496)* *“Although important to increase chances of organ donation I think it is also not to emotionally impact family members who are already experiencing such trauma by not getting consent.” (P495)* *“The first statement feels like a desecration of the body when they haven't been granted permission to do so. More asking forgiveness than permission.” (P196)*
Specific instructions	Priority is ensuring donation takes place	*“If I decide I want it done, then I'd rather it was simply done than have to have anyone waste time following my inexpert instructions.” (P33)* *“I'm happy for my organs to be donated no matter what circumstance. I don't need to write more detailed instructions. Instructions may also delay organ donation if the health care workers find them ambiguous or unclear.” (P99)* *“I would categorically say that while my family should have some time to take things in, it should not be at the risk of making organ donation unviable. I have made it very clear to my family about my feelings but I know if something happens they will still find it difficult. Perhaps if I had the option of writing them a letter that could be read to them in the event of something happening that may make the process easier for all.” (P297)*
	To alleviate family distress	*“It would be clear to the family what my wishes were and make it easier for them to make the final decision.” (P299)* *“I think its the most important to consider my families feelings.” (P90)* *“I would like to be clear on the instructions so as not to cause any further distress to my loved ones during this difficult process.” (P481)* *“To make sure the process works smoothly and not to cause further problems for my family.” (P51)*
	Specific religious beliefs or certain organs	*“I think I would write specific instructions, perhaps about what organs can be retrieved and under what conditions because my family might not feel comfortable with certain organs being taken.” (P126)* *“I wouldn't want to have anything removed unless I have written clear instructions and my relatives are on board. I have a fear of my eyes being removed and therefore would want the option to say that I do not agree to this. I would also want to make sure that my relatives were 100% happy with my plans and that they were satisfied nothing further could be done for me.” (P149)* *“I only want to donate organs that I would be able to live without, that is, one kidney as my spiritual beliefs state that you use these in your next life.” (P38)*
	Maintaining autonomy	*“Because it gives us more autonomy over our bodies after death.” (P183)* *“I am already a registered donor, so whether it would upset relatives or not, that is not my concern. My wishes need to be adhered to when I have no voice.” (P355)* *“I believe it's my body and my choice. My family understands the value of life. If after death, my organ was needed to save another life, they wouldn't hesitate to give consent.” (P374)*
	Not informed enough	*“I don't really know what i would write because i don't know what options i could specify.” (P104)* *“I didn't know that tubes had to be inserted into the leg before this study, think I would want more information on how it happens to make a better decision.” (P102)* *“I'm in favour of organ donation, but this survey has given me information I wasn't aware of. So I need to think again and let my family know. It's not as easy as I thought especially as I've lost family members and don't know now how I'd feel.” (P188)* *“It is hard to consider all possible scenarios. So I prefer a simple consent to donate without any specific instructions.” (P221)*

### 3.5 Views on Consent

#### 3.5.1 Consent – Team and Content of Conversation

In general, when asked ‘Who is best placed to have a conversation about organ donation, treating doctors or the specialist organ donation team?’ participants wanted to have conversations with both sets of clinicians: “*both have different viewpoints, both would be helpful” (P346)*. Participants felt that the treating doctors were more likely to have built up a relationship with them: “*the doctors who have been treating my family would be familiar to me, so it would be more comforting” (P421).* Whereas they felt that the specialist organ donation team would be more informed about the procedure and better able to take consent: *“I imagine there would be a lot of questions that the specialist would be best placed to answer” (P303)*. A tiny minority voiced concerns about a conflict of interest of the treating team initiating the conversation: *“I don't want to think that the treatment team had anything on their minds except saving my loved one. No suspicions of conflict of interest” (P391).* Others voiced concerns about conflicts of interest within the specialist team: *“I think the doctor has more of an interest in treating my relative, whereas the organ donation team have more of an interest in donating the organs from my relative – I feel the doctor is more on my side” (P126)*. Participants were almost universal in noting the significance of having a good conversation ‐ *“I would want to be as informed and ‘in the loop’ as possible” (P329)* ‐ to aid their understanding of the process: *“I would want as much relevant information as possible to be provided so as I feel fully informed and aware of the procedures that will follow” (P81).* They highlighted the value of doing so in a sensitive, compassionate, and open manner: *“I think it's important to keep communication honest and clear” (p136)*.

#### 3.5.2 Consent – Timing and Organ Preservation

Almost all (93%) participants thought they would need less than 4 h to think about whether their family member would want to donate their organs, with the majority (69%) thinking they would need less than an hour.

We intentionally provided detail about the cannulation and organ perfusion that is required (see Box 2, scenario 3) in order to ask further questions about this process, starting with: ‘What is more important: organ preservation or family consent?’

**FIGURE 3:**
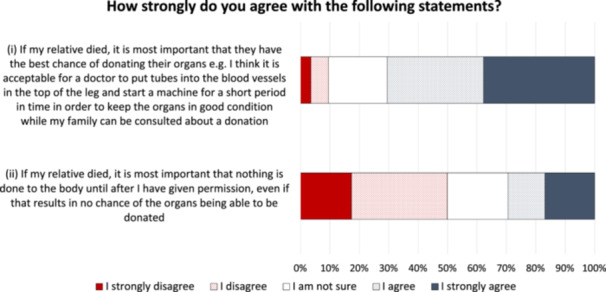
Prioritisation of preserving the chance to donate vs. obtaining family consent and maintaining bodily integrity (*n* = 504).

**FIGURE 4:**
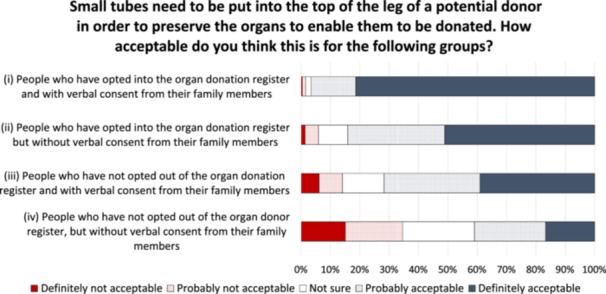
Acceptability of commencing organ preservation techniques with different levels of consent from family members and the potential donor (*n* = 504).

The majority of participants thought it was more important that their relative had the best chance of donating organs (with the explicit interventions listed) than that nothing was done to the body until after permission had been granted (see Figure 3). Participants wanted to respect their relative's wishes to donate: “*if I were the relative I'd be devastated if the chance to donate was missed just because in my absence no‐one quite liked to do the necessary actions to make the choice possible” (P379).* They also demonstrated broader altruistic intent: *“I wouldn't want to find out that someone else would not be able to benefit because time had to be taken to get my permission. This would feel like the death was completely for nothing rather than there being some benefit to someone else” (P271)*. Many noted the time constraint and believed that the intervention would be helpful for the family: “*it gives precious time to be able to have a discussion with the family in a sympathetic and caring manner. Allowing them time to grieve. The chances are the patient has already got lines and tubes attached so one more isn't going to be any more invasive” (P311)*. Others were pragmatically altruistic about the approach: *“the first [tubes into blood vessels] doesn't actually affect the organs, and is simply a practical action in order to ‘bide time’ to enable the decision to be made. Without this, there would be many fewer viable organs retrievable for donation” (P305)*. Those who were against often cited the importance of permission or consent: *“I think it's important to have permission otherwise you may feel that your loved one had been violated” (P136)*. The majority of participants found it acceptable for the cannulation to occur in order to facilitate organ preservation in all circumstances, with more support in circumstances where a patient had opted in, or verbal consent had been obtained (see Figure 4).

#### 3.5.3 Prospective Consent

When asked if they would like to be able to write specific instructions about donation (and given some examples, e.g. a patient may wish to donate but not if it were to distress their loved ones; or a patient may wish to donate, but not if it required specified interventions) 49% said no, 19% were not sure, and 32% said yes.

Of those who said no, the strongest theme was wanting to ensure organ donation takes place: *“it is my decision so would want to go ahead in any scenario” (P3)*. Some people wrote they would want to be able to write instructions for the same reason: *“I believe just opting into organ donation could still cause hiccups when a family is extremely against organ donation. If I could write a note saying ‘opt into organ donation – NO MATTER WHAT WITHOUT EXCEPTION’ – I likely would” (P78)*. Some were concerned about the possible negative impacts of specific instructions on family: *“I think this idea opens a can of worms and the organs should just be taken behind closed doors so my family doesn't have to think about it” (P13).* Others were concerned about time: “*I don't want to go into detail, just get the donation done” (P28)*. Some participants (who responded either ‘no’ or ‘don't know’) felt they were not informed enough: *“I don't think I know enough to write out specific instructions. I think my loved ones know that if it's my decision they will honour it” (P16)*. Of those who did want to leave specific instructions, the most common theme was to make things easier and less distressing for the family members: *“it takes the burden away from the family if you have already made the decisions before hand and they know what you have decided” (P327)*. Other reasons included specific religious beliefs, or desires not to have certain organs – for example, eyes – donated. Others mentioned the importance of being able to remain autonomous: *“because bodily autonomy is still a thing, even if I'm dead” (P205)*. Finally, despite us being explicit in our question about specific instructions being able to relate to procedures only two participants of the 505 mentioned this – one to say that they needed to find out more, and one said: *“I wasn't aware of the tube‐in‐the‐leg – I'm now adding something to the ‘when I die’ instructions to cover that, and anything else necessary to make donation possible” (P331)*.

## 4 Discussion

### 4.1 Key Findings

This study offers an evaluation of public attitudes toward organ donation practices, including the use of perfusion technologies and donation after out‐of‐hospital cardiac arrest. Key findings reveal strong support for all forms of donation in the UK, including uncontrolled donation after circulatory death (uDCD), consistent with Bruce et al.'s findings among Emergency Department attendees [31]. Interestingly, most participants believed uDCD was already part of current UK practice, despite this not being the case [32], which may have contributed to their acceptance.

Previous studies have indicated public acceptability of organ‐preserving technologies [20, 33, 34], and our qualitative data help explain these findings. We observed broad acceptance of NRP, with participants citing altruistic motives and the benefit of giving families and medical teams more time while respecting the donor's wishes. A better understanding of public views of NRP can contribute significantly to the ongoing ethical debate surrounding NRP. A recent study confirmed the absence of cerebral blood flow during NRP [35], addressing concerns about the potential for NRP to compromise the principle of permanence of circulatory death, and studies of the effects of CPR and NRP continue to be published [36, 37, 38]. It is notable that ethical discussion about the evolving role of regional perfusion technology continues [39, 40]. There are two particularly relevant findings in this context. First, participants supported proceeding with NRP based on verbal consent. A pilot is currently being undertaken with this protocol [41]; it is being evaluated both in terms of feasibility and patient and clinician experience. Second, although a small minority expressed unease about perfusing organs, no participants questioned whether NRP might invalidate death diagnoses.

Some participants expressed discomfort with specific aspects of donation, which supports critiques that the organ donor register may be too simplistic. It has been argued that people should be offered more detailed choices at the time of registration [42]. In our study, responses on this proposal were mixed. While most participants preferred not to provide additional instructions, some advocated for a voluntary ‘white space’ to allow optional clarification. We agree that this could offer useful context to families and clinicians, without adding burdens to registrants who prefer simplicity.

Previous research has shown that public understanding of donation practices is often limited [16, 21, 43]. Our findings confirm this, reinforcing the need for continued public education. While public engagement is improving – 39.8% of individuals surveyed by National Health Service Blood and Transplant (NHSBT) have opted in and 3.8% have opted out – over half of the UK population still has no documented preference [44]. Given our findings of how important it is to know the deceased wishes, efforts need to be renewed to rectify this. Making information available for those who want it and providing the option to express detailed wishes would promote autonomy and support clearer decision‐making by families and healthcare teams.

### 4.2 Strengths and Limitations

This was an online questionnaire‐based study of a representative UK sample by age, gender, and ethnicity; participants' religion and experience of family discussions about deceased organ donation were unknown. Like any written questionnaire, participation required a good level of English literacy and visual acuity; our survey was also limited by only recruiting those with digital literacy and internet connectivity and so the inherent selection bias for these groups needs to be considered. Purposive interview‐based research will be necessary to capture the views of those excluded by these requirements. Additionally, participants responded to hypothetical scenarios; future studies involving families approached for donation in real contexts will help assess how well these views align with real‐life decisions. Strengths of this study include the large, representative sample (whose responses were consistent with large‐scale national assessments of willingness to donate more generally [30]) and a robust analysis of qualitative data, offering insight into the quantitative results.

In summary, we demonstrated widespread support for organ donation in different circumstances, including uDCD, which many participants incorrectly assumed already occurs in the UK. There is also substantial acceptance of NRP and a willingness to donate following sudden irreversible cardiac arrest. Few participants raised concerns about death determination in this context.

Future research should explore the views of bereaved families approached about uDCD and trial and evaluate the use of ‘white space’ in an organ donation form to write more specific wishes if desired. There is still a need for increased public engagement about organ donation, and we have highlighted some misunderstandings which need to be addressed. Our findings also suggest that there is unlikely to be significant public objection to the introduction of Organ Donation After Sudden Irreversible Cardiac Arrest (ODASICA), which we propose as a more accurate abbreviation than uDCD [14]. We propose that it should be introduced and evaluated more widely; many people would welcome expanded donation opportunities to increase life‐saving transplants for patients with end‐stage organ failure.

## Author Contributions

**Weiyi Tan:** formal analysis, investigation, methodology, original draft writing, review and editing. **Janet Cruse:** methodology, formal analysis, review and editing. **Dominic Summers:** formal analysis, funding aquisition, investigation, methodology, supervision, review and editing. **Zoë Fritz:** conceptulaisation, formal analysis, funding aquisition, investigation, methodology, project administration, supervision, original draft writing review and editing.

## Ethics Statement

Ethics approvals were received from the Cambridge University Psychology Research Ethics Committee (PRE.2024.015).

## Conflicts of Interest

The authors declare no conflicts of interest.

## Data Availability

The data that support the findings of this study are available from the corresponding author upon reasonable request.
